# 2,2-Dimethyl-5-{[(4-nitro­phen­yl)amino]­methyl­idene}-1,3-dioxane-4,6-dione

**DOI:** 10.1107/S1600536811001103

**Published:** 2011-01-15

**Authors:** Ying-Hong Yang, Zi-Cheng Li, You-Fu Luo

**Affiliations:** a Department of Pharmaceutical and Bioengineering, School of Chemical Engineering, Sichuan University, Chengdu 610065, People’s Republic of China; b State Key Laboratory of Biotherapy, West China Hospital, Sichuan University, Chengdu 610041, People’s Republic of China

## Abstract

In the title compound, C_13_H_12_N_2_O_6_, the dihedral angle between the benzene ring and the amino­methyl­ene unit is 5.42 (16)°, while the angle between the amino­methyl­ene unit and the dioxane ring is 3.06 (43)°. The dioxane ring shows a half-boat conformation, in which the C atom between the dioxane ring O atoms is 0.464 (10) Å out of the plane. An intra­molecular N—H⋯O hydrogen bond stabilizes the mol­ecular conformation. In the crystal, a three-dimensional framework is built up *via* inter­molecular N—H⋯O hydrogen bonds.

## Related literature

For the synthesis and biological activity of related compounds, see: Cassis *et al.* (1985 ▶); Griera *et al.* (1997 ▶); Darque *et al.* (2009 ▶).
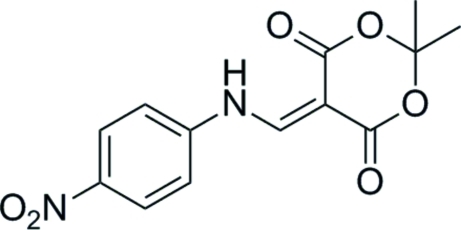


## Experimental

### 

#### Crystal data

C_13_H_12_N_2_O_6_
*M* *_r_* = 292.25
Monoclinic, 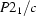

*a* = 12.2822 (8) Å
*b* = 12.2762 (7) Å
*c* = 9.2760 (6) Å
β = 106.636 (7)°
*V* = 1340.08 (15) Å^3^
*Z* = 4
Mo *K*α radiation
μ = 0.12 mm^−1^
*T* = 293 K
0.22 × 0.15 × 0.10 mm

#### Data collection

Oxford Diffraction Xcalibur Eos diffractometer
Absorption correction: multi-scan (*CrysAlis PRO*; Oxford Diffraction, 2010 ▶) *T*_min_ = 0.933, *T*_max_ = 1.0
6063 measured reflections
2741 independent reflections
1432 reflections with *I* > 2σ(*I*)
*R*_int_ = 0.031

#### Refinement

*R*[*F* ^2^ > 2σ(*F* ^2^)] = 0.052
*wR*(*F* ^2^) = 0.126
*S* = 1.00
2741 reflections
192 parameters
H-atom parameters constrained
Δρ_max_ = 0.17 e Å^−3^
Δρ_min_ = −0.15 e Å^−3^

### 

Data collection: *CrysAlis PRO* (Oxford Diffraction, 2010 ▶); cell refinement: *CrysAlis PRO*; data reduction: *CrysAlis PRO*; program(s) used to solve structure: *SHELXS97* (Sheldrick, 2008 ▶); program(s) used to refine structure: *SHELXL97* (Sheldrick, 2008 ▶); molecular graphics: *OLEX2* (Dolomanov, 2009 ▶); software used to prepare material for publication: *OLEX2*.

## Supplementary Material

Crystal structure: contains datablocks I, global. DOI: 10.1107/S1600536811001103/pb2051sup1.cif

Structure factors: contains datablocks I. DOI: 10.1107/S1600536811001103/pb2051Isup2.hkl

Additional supplementary materials:  crystallographic information; 3D view; checkCIF report

## Figures and Tables

**Table 1:** Hydrogen-bond geometry (Å, °)

*D*—H⋯*A*	*D*—H	H⋯*A*	*D*⋯*A*	*D*—H⋯*A*
N2—H2⋯O3^i^	0.86	2.65	3.411 (3)	148
N2—H2⋯O4	0.86	2.15	2.771 (2)	129

Symmetry code: (i) 
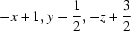
.

**Fig. 1.**
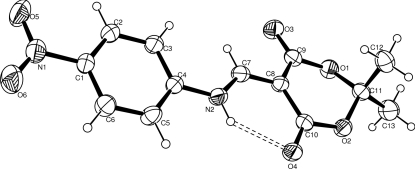
The molecular structure of the title compound, with displacement ellipsoids drawn at the 30% probability level. The intramolecular hydrogen bond is shown as a dashed line.

**Fig. 2.**
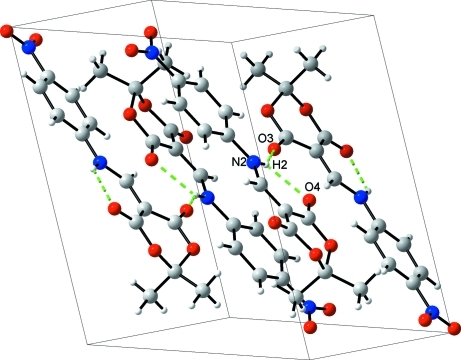
Crystal packing of the title compound, showing the intermolecular hydrogen bonds as dashed lines.

## supplementary crystallographic information

### Comment

The 4(1*H*)quinolone are of great importance owing to their wide biological properties (Griera *et al.*, 1997; Darque *et al.*, 2009). 2,2-Dimethyl-5-{[(4-nitrophenyl)amino]methylene}-1,3-dioxane-4,6-dione is the key intermediate which can be used to synthesize the 4(1*H*)quinolone derivatives by thermolysis (Cassis *et al.*, 1985). The title compound is approximately planar, the dihedral angle between the benzene ring and the aminomethylene unit is 5.42 (16), while the angle between the aminomethylene unit and the dioxane ring is 3.06 (43)°. Besides, The dioxane ring shows a half-boat conformation, in which the C atom between the dioxane ring O atoms is 0.4639 (99) A ° out of the plane.The intramolecular N—H···O hydrogen bond which involving the NH H atom and the adjacent dioxane carbonyl O atom can stabilize the planar conformation of the molecule, and the three-dimensional framework is built *via* intermolecular N—H···O hydrogen bond and weak *π*–*π* stacking interactions with a centroid–centroid distance of 5.1837 (14) Å.

### Experimental

A solution of 2,2–dimethyl–1,3–dioxane–4,6–dione (1.44 g, 10 mmol) and triethoxymethane (1.78 g, 12 mmol) was heated to reflux for 2.5 h, then the 4-nitroaniline(1.38 g, 10 mmol) was added into the above solution. The mixture was heated under reflux for another 7 h. The precipitate that formed was filtered off and recrystallized from ethanol, giving the title compound. Crystals suitable for X-ray analysis were obtained by slow evaporation from a solution of ethanol.

### Refinement

The H atom of N2 was located in a difference map and refined isotropically. The reminaing H atoms were positioned geometrically (C—H = 0.93–0.96 Å) and refined using a riding model, with U*_iso_*(H) = 1.2 or 1.5U*_eq_*(C).

### Crystal data

**Table:**

C_13_H_12_N_2_O_6_	*F*(000) = 608
*M**_r_* = 292.25	*D*_x_ = 1.449 Mg m^−^^3^
Monoclinic, *P*2_1_/*c*	Mo *K*α radiation, λ = 0.7107 Å
Hall symbol: -P 2ybc	Cell parameters from 1825 reflections
*a* = 12.2822 (8) Å	θ = 3.0–29.1°
*b* = 12.2762 (7) Å	µ = 0.12 mm^−^^1^
*c* = 9.2760 (6) Å	*T* = 293 K
β = 106.636 (7)°	Block, colorless
*V* = 1340.08 (15) Å^3^	0.22 × 0.15 × 0.10 mm
*Z* = 4	

### Data collection

**Table:**

Oxford Diffraction Xcalibur Eos diffractometer	2741 independent reflections
Radiation source: fine-focus sealed tube	1432 reflections with *I* > 2σ(*I*)
graphite	*R*_int_ = 0.031
Detector resolution: 16.0874 pixels mm^-1^	θ_max_ = 26.4°, θ_min_ = 3.0°
ω scans	*h* = −15→14
Absorption correction: multi-scan (*CrysAlis PRO*; Oxford Diffraction, 2010)	*k* = −15→14
*T*_min_ = 0.933, *T*_max_ = 1.0	*l* = −11→11
6063 measured reflections	

### Refinement

**Table:**

Refinement on *F*^2^	Primary atom site location: structure-invariant direct methods
Least-squares matrix: full	Secondary atom site location: difference Fourier map
*R*[*F*^2^ > 2σ(*F*^2^)] = 0.052	Hydrogen site location: inferred from neighbouring sites
*wR*(*F*^2^) = 0.126	H-atom parameters constrained
*S* = 1.00	*w* = 1/[σ^2^(*F*_o_^2^) + (0.0459*P*)^2^] where *P* = (*F*_o_^2^ + 2*F*_c_^2^)/3
2741 reflections	(Δ/σ)_max_ < 0.001
192 parameters	Δρ_max_ = 0.17 e Å^−^^3^
0 restraints	Δρ_min_ = −0.15 e Å^−^^3^

### Special details

**Table:**

Experimental. CrysAlisPro, Oxford Diffraction Ltd., Version 1.171.34.40 (release 27-08-2010 CrysAlis171 .NET) (compiled Aug 27 2010,11:50:40) Empirical absorption correction using spherical harmonics, implemented in SCALE3 ABSPACK scaling algorithm.
Geometry. All e.s.d.'s (except the e.s.d. in the dihedral angle between two l.s. planes) are estimated using the full covariance matrix. The cell e.s.d.'s are taken into account individually in the estimation of e.s.d.'s in distances, angles and torsion angles; correlations between e.s.d.'s in cell parameters are only used when they are defined by crystal symmetry. An approximate (isotropic) treatment of cell e.s.d.'s is used for estimating e.s.d.'s involving l.s. planes.
Refinement. Refinement of *F*^2^ against ALL reflections. The weighted *R*-factor *wR* and goodness of fit *S* are based on *F*^2^, conventional *R*-factors *R* are based on *F*, with *F* set to zero for negative *F*^2^. The threshold expression of *F*^2^ > σ(*F*^2^) is used only for calculating *R*-factors(gt) *etc*. and is not relevant to the choice of reflections for refinement. *R*-factors based on *F*^2^ are statistically about twice as large as those based on *F*, and *R*- factors based on ALL data will be even larger.

### Fractional atomic coordinates and isotropic or equivalent isotropic displacement parameters (Å^2^)

**Table:**

	*x*	*y*	*z*	*U*_iso_*/*U*_eq_	
O1	0.69558 (14)	0.20994 (13)	0.55007 (17)	0.0590 (5)	
O2	0.68603 (13)	0.02148 (12)	0.59936 (18)	0.0621 (5)	
O3	0.58804 (16)	0.33063 (14)	0.6199 (2)	0.0731 (6)	
O4	0.55320 (14)	−0.04417 (12)	0.69224 (19)	0.0669 (5)	
O5	0.07063 (17)	0.30379 (18)	1.0992 (2)	0.0965 (7)	
O6	0.02953 (19)	0.13441 (18)	1.1075 (2)	0.0978 (7)	
N1	0.08472 (19)	0.2076 (2)	1.0746 (2)	0.0682 (6)	
N2	0.42612 (16)	0.10150 (15)	0.80560 (19)	0.0532 (5)	
H2	0.4389	0.0332	0.7979	0.064*	
C1	0.1735 (2)	0.1808 (2)	1.0033 (3)	0.0512 (6)	
C2	0.2364 (2)	0.2628 (2)	0.9673 (3)	0.0610 (7)	
H2A	0.2225	0.3349	0.9872	0.073*	
C3	0.3200 (2)	0.23774 (19)	0.9015 (3)	0.0599 (7)	
H3	0.3625	0.2931	0.8752	0.072*	
C4	0.3412 (2)	0.13024 (18)	0.8742 (2)	0.0475 (6)	
C5	0.2780 (2)	0.04875 (19)	0.9127 (2)	0.0578 (7)	
H5	0.2930	−0.0237	0.8959	0.069*	
C6	0.1926 (2)	0.0739 (2)	0.9760 (3)	0.0619 (7)	
H6	0.1485	0.0191	0.9998	0.074*	
C7	0.4875 (2)	0.17060 (19)	0.7523 (2)	0.0519 (6)	
H7	0.4728	0.2442	0.7615	0.062*	
C8	0.5701 (2)	0.14631 (17)	0.6851 (2)	0.0473 (6)	
C9	0.6166 (2)	0.2361 (2)	0.6219 (3)	0.0525 (6)	
C10	0.60071 (19)	0.03607 (19)	0.6628 (2)	0.0511 (6)	
C11	0.7596 (2)	0.11232 (19)	0.5931 (3)	0.0532 (6)	
C12	0.8456 (2)	0.1284 (2)	0.7444 (3)	0.0690 (8)	
H12A	0.8068	0.1446	0.8183	0.104*	
H12C	0.8950	0.1878	0.7387	0.104*	
H12B	0.8896	0.0632	0.7726	0.104*	
C13	0.8114 (2)	0.0867 (2)	0.4690 (3)	0.0759 (8)	
H13B	0.8543	0.0204	0.4920	0.114*	
H13C	0.8607	0.1452	0.4593	0.114*	
H13A	0.7523	0.0782	0.3762	0.114*	

### Atomic displacement parameters (Å^2^)

**Table:**

	*U*^11^	*U*^22^	*U*^33^	*U*^12^	*U*^13^	*U*^23^
O1	0.0627 (12)	0.0520 (10)	0.0669 (11)	−0.0033 (9)	0.0258 (10)	0.0107 (8)
O2	0.0610 (11)	0.0457 (10)	0.0923 (12)	−0.0069 (8)	0.0423 (10)	−0.0037 (9)
O3	0.0796 (14)	0.0420 (10)	0.1021 (14)	−0.0003 (10)	0.0332 (11)	0.0071 (10)
O4	0.0672 (12)	0.0411 (10)	0.1041 (14)	−0.0106 (9)	0.0433 (11)	−0.0009 (9)
O5	0.0895 (16)	0.0796 (15)	0.1366 (19)	0.0217 (12)	0.0585 (15)	−0.0083 (13)
O6	0.0944 (17)	0.0955 (16)	0.1287 (18)	−0.0093 (13)	0.0723 (15)	0.0001 (13)
N1	0.0586 (16)	0.0776 (17)	0.0710 (15)	0.0078 (14)	0.0227 (13)	−0.0020 (14)
N2	0.0587 (14)	0.0437 (11)	0.0612 (12)	−0.0031 (10)	0.0239 (11)	−0.0054 (10)
C1	0.0471 (15)	0.0550 (16)	0.0535 (15)	0.0051 (13)	0.0174 (12)	−0.0008 (12)
C2	0.0594 (17)	0.0466 (15)	0.0818 (18)	0.0016 (13)	0.0277 (15)	−0.0086 (14)
C3	0.0608 (17)	0.0451 (15)	0.0818 (18)	−0.0078 (13)	0.0331 (15)	−0.0046 (13)
C4	0.0484 (15)	0.0463 (14)	0.0487 (14)	0.0026 (12)	0.0153 (12)	−0.0019 (11)
C5	0.0762 (19)	0.0415 (14)	0.0645 (16)	0.0012 (13)	0.0342 (15)	0.0017 (12)
C6	0.0730 (19)	0.0531 (17)	0.0698 (17)	−0.0020 (14)	0.0369 (15)	0.0070 (13)
C7	0.0546 (16)	0.0451 (14)	0.0541 (15)	−0.0042 (12)	0.0123 (13)	−0.0046 (12)
C8	0.0506 (15)	0.0378 (13)	0.0560 (15)	−0.0043 (11)	0.0192 (13)	0.0001 (11)
C9	0.0492 (16)	0.0509 (16)	0.0528 (15)	−0.0054 (13)	0.0072 (12)	0.0013 (13)
C10	0.0473 (15)	0.0470 (15)	0.0599 (15)	−0.0066 (12)	0.0169 (13)	−0.0002 (12)
C11	0.0536 (16)	0.0483 (15)	0.0624 (16)	−0.0090 (13)	0.0238 (14)	0.0034 (13)
C12	0.0577 (18)	0.0740 (18)	0.0759 (18)	−0.0065 (15)	0.0199 (15)	0.0105 (15)
C13	0.078 (2)	0.0794 (19)	0.0812 (18)	−0.0113 (17)	0.0400 (17)	−0.0039 (16)

### Geometric parameters (Å, °)

**Table:**

O1—C9	1.363 (3)	C3—C4	1.382 (3)
O1—C11	1.426 (3)	C4—C5	1.375 (3)
O2—C10	1.353 (2)	C5—H5	0.9300
O2—C11	1.447 (3)	C5—C6	1.375 (3)
O3—C9	1.211 (3)	C6—H6	0.9300
O4—C10	1.215 (2)	C7—H7	0.9300
O5—N1	1.224 (3)	C7—C8	1.366 (3)
O6—N1	1.216 (3)	C8—C9	1.442 (3)
N1—C1	1.465 (3)	C8—C10	1.435 (3)
N2—H2	0.8600	C11—C12	1.508 (3)
N2—C4	1.414 (3)	C11—C13	1.500 (3)
N2—C7	1.322 (3)	C12—H12A	0.9600
C1—C2	1.367 (3)	C12—H12C	0.9600
C1—C6	1.370 (3)	C12—H12B	0.9600
C2—H2A	0.9300	C13—H13B	0.9600
C2—C3	1.372 (3)	C13—H13C	0.9600
C3—H3	0.9300	C13—H13A	0.9600
			
O1—C9—C8	116.1 (2)	C4—C5—H5	119.9
O1—C11—O2	110.98 (19)	C4—C5—C6	120.2 (2)
O1—C11—C12	109.5 (2)	C5—C4—N2	118.7 (2)
O1—C11—C13	106.38 (19)	C5—C4—C3	119.8 (2)
O2—C10—C8	117.1 (2)	C5—C6—H6	120.4
O2—C11—C12	110.04 (19)	C6—C1—N1	119.3 (2)
O2—C11—C13	106.03 (19)	C6—C5—H5	119.9
O3—C9—O1	117.5 (2)	C7—N2—H2	117.2
O3—C9—C8	126.3 (2)	C7—N2—C4	125.6 (2)
O4—C10—O2	118.2 (2)	C7—C8—C9	116.8 (2)
O4—C10—C8	124.7 (2)	C7—C8—C10	122.1 (2)
O5—N1—C1	117.7 (2)	C8—C7—H7	116.3
O6—N1—O5	123.2 (2)	C9—O1—C11	118.27 (17)
O6—N1—C1	119.2 (2)	C10—O2—C11	119.07 (18)
N2—C7—H7	116.3	C10—C8—C9	120.7 (2)
N2—C7—C8	127.4 (2)	C11—C12—H12A	109.5
C1—C2—H2A	120.3	C11—C12—H12C	109.5
C1—C2—C3	119.4 (2)	C11—C12—H12B	109.5
C1—C6—C5	119.2 (2)	C11—C13—H13B	109.5
C1—C6—H6	120.4	C11—C13—H13C	109.5
C2—C1—N1	119.4 (2)	C11—C13—H13A	109.5
C2—C1—C6	121.4 (2)	H12A—C12—H12C	109.5
C2—C3—H3	120.0	H12A—C12—H12B	109.5
C2—C3—C4	120.0 (2)	H12C—C12—H12B	109.5
C3—C2—H2A	120.3	C13—C11—C12	113.8 (2)
C3—C4—N2	121.5 (2)	H13B—C13—H13C	109.5
C4—N2—H2	117.2	H13B—C13—H13A	109.5
C4—C3—H3	120.0	H13C—C13—H13A	109.5

### Hydrogen-bond geometry (Å, °)

**Table:**

*D*—H···*A*	*D*—H	H···*A*	*D*···*A*	*D*—H···*A*
N2—H2···O3^i^	0.86	2.65	3.411 (3)	148
N2—H2···O4	0.86	2.15	2.771 (2)	129

Symmetry codes: (i) −*x*+1, *y*−1/2, −*z*+3/2.
